# Supplementary material for: A machine learning approach to brain epigenetic analysis reveals kinases associated with Alzheimer’s disease
Source: Nat Commun. 2021 Jul 22;12:4472. doi: 10.1038/s41467-021-24710-8 (PMC8298578; doi:10.1038/s41467-021-24710-8)
Supplement: Supplementary file 1 — Supplementary Information [file 41467_2021_24710_MOESM1_ESM.pdf]

Supplementary Information for: A machine learning approach leveraging epigenome associations identifies new Alzheimer's disease loci

Huang et al.

This supplementary Information contains Supplementary Figures 1 to 6 and Supplementary Tables 1 to 10.

Supplementary Figures

Supplementary Figure 1. Comparison of methylation coverage between Illumina 450K methylation array and EWASplus. **a.** The density of CpGs covered by Illumina 450K methylation array. **b.** The density of CpGs covered by EWASplus. The figure legend for both subplots has the same color bar scale. The darker red indicates a higher CpG density and the darker green means a lower CpG density.

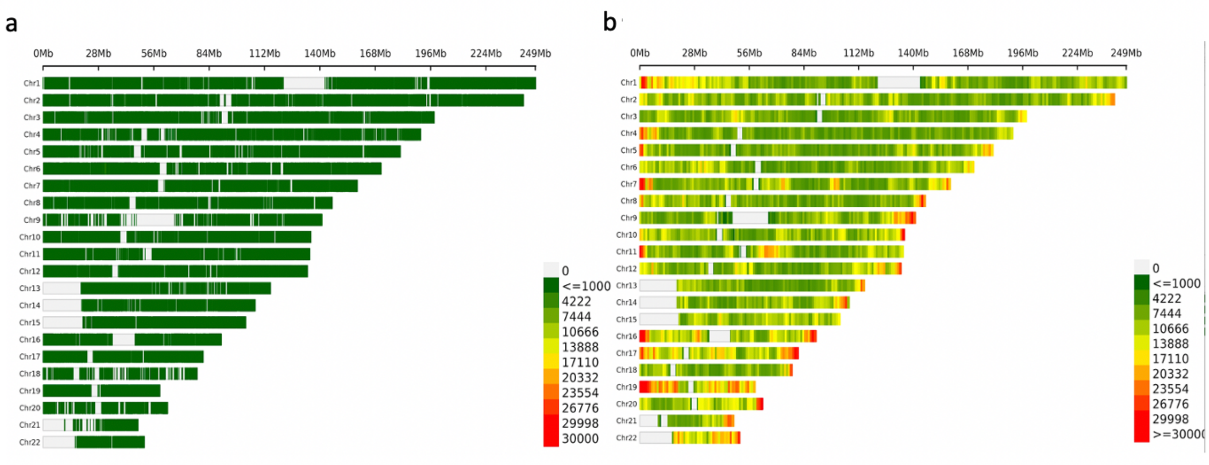

Supplementary Figure 2. The box plots of the prediction scores for CpGs on 450K array. In box plots, the central mark indicates the median, and the lower and upper edges of the box correspond to the 25th and 75th percentiles. The maximum whisker lengths are specified as 1.5 times the interquartile range (IQR) and outliers are marked using red dots. The CpGs on 450K array are grouped into four groups: 1) the positive and 2) the negative CpGs included in the model training and 3) near positive, 4) randomly chosen negative CpGs, which are matched for the exact same number with the first two groups, respectively. "Near positive" and randomly chose negative CpGs are not included in the training set, hence, they can better reflect the prediction scores distribution for the "unseen" positive and negative loci with less overfitting tendency. The "near positive" CpGs are those with the significance levels that are just above the p-value threshold of distinguishing positive loci. The p-value threshold for selecting positive loci varies by different traits based on the distribution of significance levels from the differential methylation association studies. We found there is a significant difference for EWASplus prediction scores between positive and negative CpGs that are not in the training sets (the middle two boxes; one-sided Wilcoxon Rank Sum test; $p = 3.64 \times 10^{-16}$ for beta-amyloid, $p = 1.38 \times 10^{-43}$ for Braak staging, $p = 3.67 \times 10^{-50}$ for CERAD, $p = 1.50 \times 10^{-27}$ for cognitive decline trajectory, $p = 5.04 \times 10^{-76}$ for global pathology, $p = 2.82 \times 10^{-99}$ for neurofibrillary tangles). Source data are provided as a Source Data file.

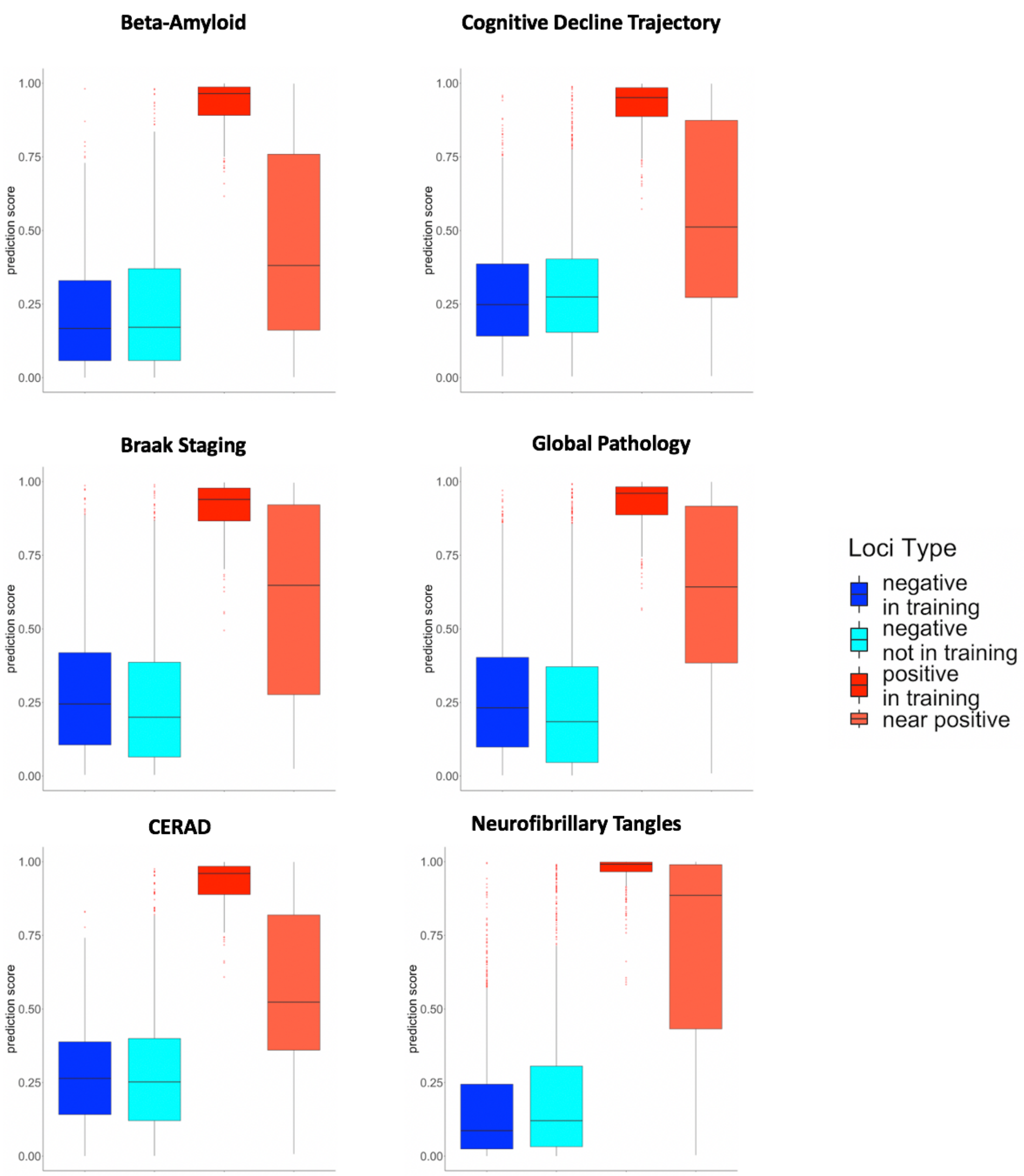
Beta-Amyloid
Cognitive Decline Trajectory
Braak Staging
Global Pathology
CERAD
Neurofibrillary Tangles
prediction score
0.00
0.25
0.50
0.75
1.00
Loci Type
negative in training
negative not in training
positive in training
near positive

Supplementary Figure 3. Performance comparison between EWASplus and adapted Zhang et al. methylation level imputation method. **a**. Receiver Operating Characteristic curves of the predictive performance of EWASplus versus adapted Zhang et al. method. **b.** Precision-Recall curves of the predictive performance of EWASplus versus adapted Zhang et al. method. Source data are provided as a Source Data file.

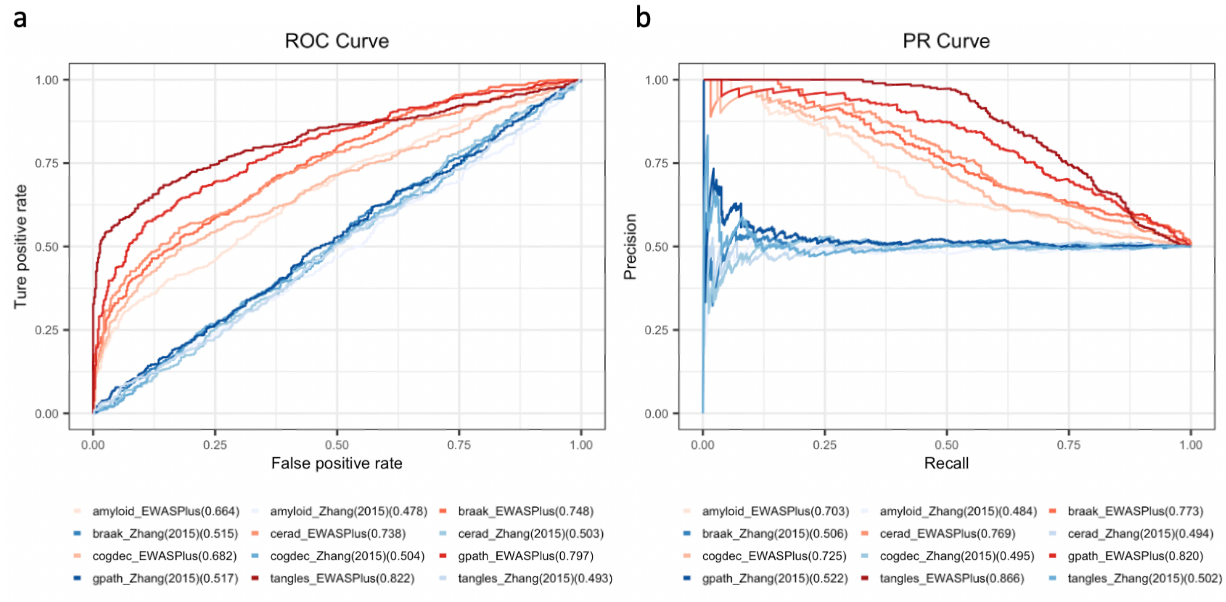

Supplementary Figure 4. a. Receiver Operating Characteristic curves of the predictive performance of EWASplus on London, Mount Sinai and Arizona cohorts. **b.** Precision-Recall curves of the predictive performance of EWASplus in London, Mount Sinai and Arizona cohorts. Source data are provided as a Source Data file.

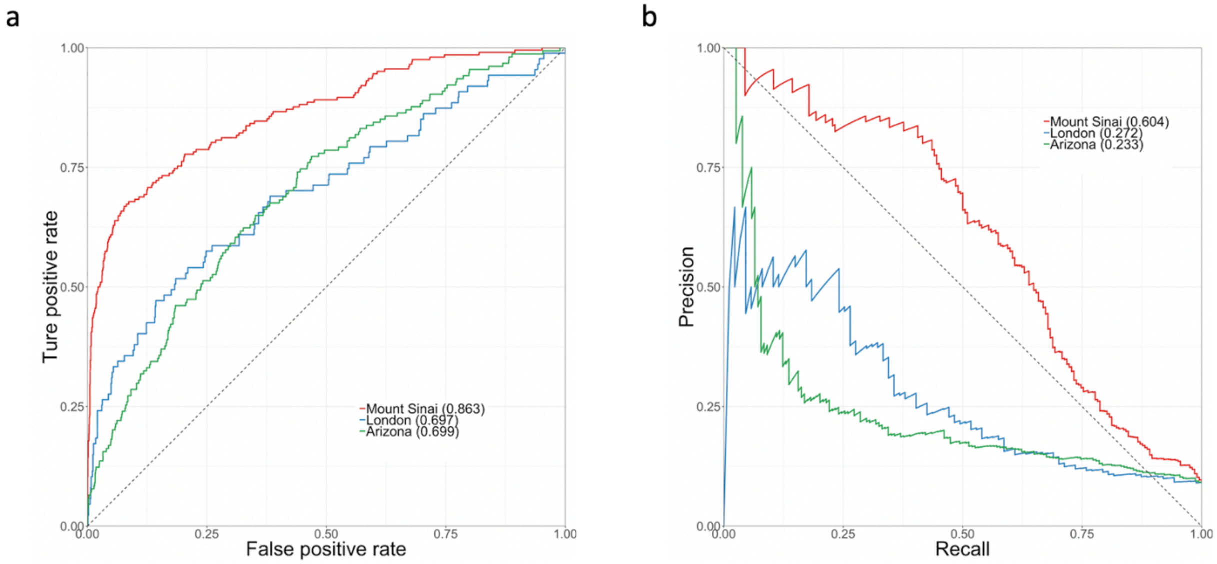

Supplementary Figure 5. The scatter plots of performance measures versus p-value threshold for significant CpGs selection of different cohorts a. AUC, **b.** AUPR, **c.** F1, **d.** precision, **e.** Recall. The link between abbreviation and the full name of the cohort are as following: RM: ROS/MAP cohort, AZ: Arizona cohort, MS: Mount Sinai cohort, LD: London cohort. Source data are provided as a Source Data file.

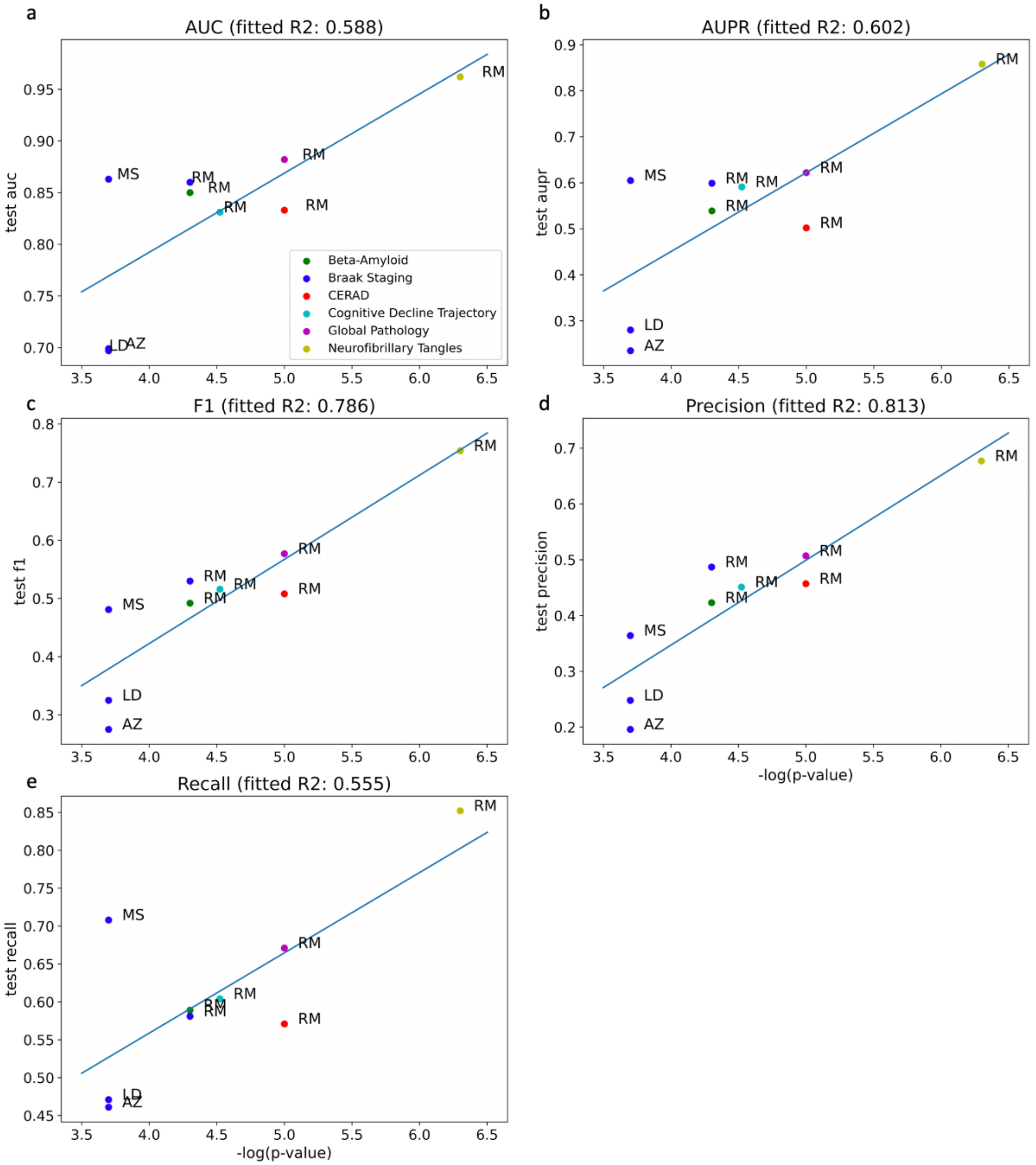

Supplementary Figure 6. Selected protein-protein interaction (PPI) networks and communities among known AD GWAS genes (n=28) and top AD EWASplus genes (n=123). The lines represent physical PPIs between proteins. The thickness of the lines is proportional to the evidence for the PPI. The black asterisk indicates genes that are known kinases.

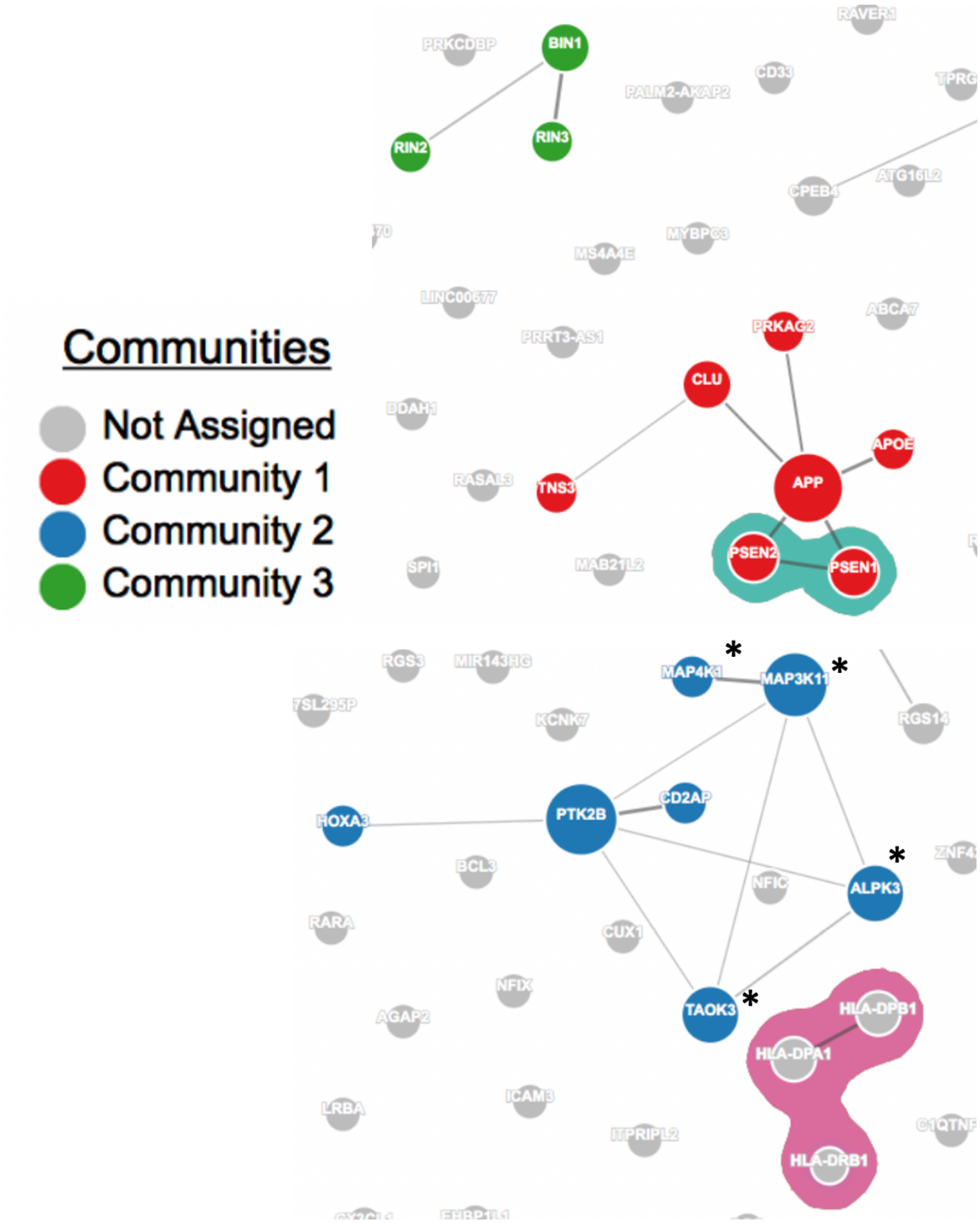
Communities
Not Assigned
Community 1
Community 2
Community 3
BIN1
RIN2
RIN3
PRKCDBP
PALM2-AKAP2
CD33
RAVER1
CPEB4
ATG16L2
MS4A4E
MYBPC3
LINC00677
PRRT3-AS1
ABCA7
PRKAG2
CLU
APOE
APP
TNS3
PSEN2
PSEN1
DDAH1
RASAL3
SPI1
MAB21L2
RGS3
MIR143HG
MAP4K1
MAP3K11
7SL295P
KCNK7
RGS14
HOXA3
PTK2B
CD2AP
BCL3
NFIC
ALPK3
RARA
CUX1
NFIX
TAOK3
HLA-DPB1
HLA-DPA1
AGAP2
LRBA
ICAM3
ITPRIPL2
HLA-DRB1
*
*
*
*

Supplementary Table 1. Performance of different combination of Machine learning models on Beta Amyloid. (with upsampling). Abbreviation used in the table: RLR - Regularized Logistic Regression, RF – Random Forest, GBDT – Gradient Boosting Decision Trees, SVM – Support Vector Machine.

	**accuracy**	**auc**	**f1**	**precision**	**recall**
**RLR**	0.7152	0.8212	0.7429	0.6771	0.8228
**RLR-RF**	0.7595	0.8439	0.7286	0.8361	0.6456
**RLR-GBDT**	0.7563	0.8449	0.7050	0.8932	0.5823
**RLR-GBDT-RF**	0.7247	0.8524	0.6390	0.9277	0.4873
**GBDT**	0.6962	0.8391	0.5752	0.9559	0.4114
**RLR-SVM-GBDT**	0.6930	0.8473	0.5611	0.9841	0.3924
**GBDT-RF**	0.6835	0.8516	0.5455	0.9677	0.3797
**RLR-SVM-GBDT-RF**	0.6804	0.8544	0.5302	1.0000	0.3608
**RLR-SVM**	0.6266	0.8280	0.4100	0.9762	0.2595
**RLR-SVM-RF**	0.6108	0.8463	0.3627	1.0000	0.2215
**SVM-GBDT-RF**	0.6013	0.8523	0.3368	1.0000	0.2025
**SVM-GBDT**	0.5981	0.8394	0.3280	1.0000	0.1962
**RF**	0.5886	0.8382	0.3085	0.9667	0.1835
**SVM**	0.5886	0.7939	0.3011	1.0000	0.1772
**SVM-RF**	0.5886	0.8403	0.3011	1.0000	0.1772

Supplementary Table 2. Performance of different combination of Machine learning models on Braak Staging. (with upsampling). Abbreviation used in the table: RLR - Regularized Logistic Regression, RF – Random Forest, GBDT – Gradient Boosting Decision Trees, SVM – Support Vector Machine.

	**accuracy**	**auc**	**f1**	**precision**	**recall**
**RLR-RF**	0.7880	0.8575	0.7568	0.8873	0.6597
**RLR**	0.7225	0.8232	0.7488	0.6840	0.8272
**RLR-GBDT**	0.7696	0.8592	0.7215	0.9120	0.5969
**RLR-GBDT-RF**	0.7592	0.8689	0.6954	0.9459	0.5497
**GBDT**	0.7539	0.8748	0.6824	0.9619	0.5288
**RLR-SVM-GBDT**	0.7461	0.8581	0.6756	0.9352	0.5288
**GBDT-RF**	0.7408	0.8825	0.6598	0.9600	0.5026
**RLR-SVM-GBDT-RF**	0.7304	0.8672	0.6485	0.9314	0.4974
**RLR-SVM-RF**	0.7147	0.8553	0.6175	0.9362	0.4607
**RLR-SVM**	0.6990	0.8333	0.5907	0.9222	0.4346
**SVM-GBDT-RF**	0.7016	0.8798	0.5870	0.9529	0.4241
**RF**	0.7042	0.8715	0.5861	0.9756	0.4188
**SVM-GBDT**	0.6780	0.8731	0.5428	0.9359	0.3822
**SVM-RF**	0.6649	0.8668	0.5152	0.9315	0.3560
**SVM**	0.6571	0.8094	0.4981	0.9286	0.3403

Supplementary Table 3. Performance of different combination of Machine learning models on CERAD. (with upsampling). Abbreviation used in the table: RLR - Regularized Logistic Regression, RF – Random Forest, GBDT – Gradient Boosting Decision Trees, SVM – Support Vector Machine.

	**accuracy**	**auc**	**f1**	**precision**	**recall**
**RLR**	0.7210	0.7987	0.7441	0.6873	0.8112
**RLR-GBDT**	0.7661	0.8464	0.7183	0.9026	0.5966
**RLR-RF**	0.7468	0.8415	0.7094	0.8324	0.6180
**RLR-GBDT-RF**	0.7210	0.8528	0.6328	0.9256	0.4807
**RLR-SVM-GBDT**	0.7039	0.8488	0.5941	0.9439	0.4335
**RLR-SVM**	0.7039	0.8206	0.5917	0.9524	0.4292
**GBDT**	0.7017	0.8604	0.5875	0.9519	0.4249
**RLR-SVM-GBDT-RF**	0.6910	0.8536	0.5663	0.9495	0.4034
**RLR-SVM-RF**	0.6845	0.8428	0.5505	0.9574	0.3863
**GBDT-RF**	0.6824	0.8486	0.5432	0.9670	0.3777
**SVM-GBDT**	0.6567	0.8626	0.4805	0.9867	0.3176
**SVM-GBDT-RF**	0.6545	0.8509	0.4756	0.9865	0.3133
**SVM**	0.6481	0.7954	0.4675	0.9600	0.3090
**SVM-RF**	0.6438	0.8340	0.4539	0.9718	0.2961
**RF**	0.6159	0.8300	0.3849	0.9655	0.2403

Supplementary Table 4. Performance of different combination of Machine learning models on Cognitive Decline Trajectory. (with upsampling). Abbreviation used in the table: RLR - Regularized Logistic Regression, RF – Random Forest, GBDT – Gradient Boosting Decision Trees, SVM – Support Vector Machine.

	**accuracy**	**auc**	**f1**	**precision**	**recall**
**RLR**	0.6982	0.7900	0.7287	0.6618	0.8108
**RLR-RF**	0.7568	0.8303	0.7286	0.8239	0.6532
**RLR-GBDT**	0.7590	0.8267	0.7147	0.8758	0.6036
**RLR-SVM-GBDT**	0.7432	0.8272	0.6667	0.9500	0.5135
**RLR-GBDT-RF**	0.7387	0.8376	0.6628	0.9344	0.5135
**RLR-SVM**	0.7297	0.8107	0.6591	0.8923	0.5225
**RLR-SVM-GBDT-RF**	0.7297	0.8375	0.6407	0.9554	0.4820
**GBDT**	0.7207	0.8563	0.6265	0.9455	0.4685
**RLR-SVM-RF**	0.7162	0.8310	0.6228	0.9286	0.4685
**GBDT-RF**	0.7140	0.8559	0.6116	0.9524	0.4505
**SVM-GBDT-RF**	0.7005	0.8551	0.5804	0.9684	0.4144
**SVM-GBDT**	0.6914	0.8537	0.5651	0.9570	0.4009
**SVM-RF**	0.6824	0.8467	0.5437	0.9655	0.3784
**SVM**	0.6779	0.8084	0.5431	0.9341	0.3829
**RF**	0.6712	0.8479	0.5197	0.9634	0.3559

Supplementary Table 5. Performance of different combination of Machine learning models on Global Pathology. (with upsampling). Abbreviation used in the table: RLR - Regularized Logistic Regression, RF – Random Forest, GBDT – Gradient Boosting Decision Trees, SVM – Support Vector Machine.

	**accuracy**	**auc**	**f1**	**precision**	**recall**
**RLR-RF**	0.8182	0.8903	0.8030	0.8760	0.7413
**RLR-GBDT**	0.8217	0.8907	0.7976	0.9220	0.7028
**RLR**	0.7465	0.8558	0.7724	0.7009	0.8601
**RLR-GBDT-RF**	0.7745	0.9006	0.7202	0.9486	0.5804
**RLR-SVM**	0.7710	0.8709	0.7183	0.9330	0.5839
**RLR-SVM-GBDT**	0.7605	0.8918	0.7002	0.9357	0.5594
**GBDT**	0.7517	0.9072	0.6758	0.9737	0.5175
**RLR-SVM-GBDT-RF**	0.7500	0.9006	0.6757	0.9613	0.5210
**RLR-SVM-RF**	0.7378	0.8912	0.6622	0.9304	0.5140
**GBDT-RF**	0.7378	0.9032	0.6495	0.9789	0.4860
**SVM-GBDT**	0.7343	0.9095	0.6449	0.9718	0.4825
**SVM-GBDT-RF**	0.7290	0.9040	0.6318	0.9852	0.4650
**SVM**	0.7010	0.8799	0.5899	0.9389	0.4301
**SVM-RF**	0.7045	0.8933	0.5888	0.9680	0.4231
**RF**	0.6888	0.8869	0.5528	0.9821	0.3846

Supplementary Table 6. Performance of different combination of Machine learning models on Neurofibrillary Tangles. (with upsampling). Abbreviation used in the table: RLR - Regularized Logistic Regression, RF – Random Forest, GBDT – Gradient Boosting Decision Trees, SVM – Support Vector Machine.

	**accuracy**	**auc**	**f1**	**precision**	**recall**
**RLR-RF**	0.9054	0.9647	0.9029	0.9269	0.8801
**RLR-GBDT**	0.8959	0.9597	0.8904	0.9404	0.8454
**RLR-GBDT-RF**	0.8975	0.9656	0.8889	0.9701	0.8202
**RLR-SVM-GBDT-RF**	0.8912	0.9657	0.8804	0.9769	0.8013
**RLR-SVM-GBDT**	0.8896	0.9602	0.8785	0.9768	0.7981
**RLR-SVM**	0.8880	0.9549	0.8774	0.9695	0.8013
**RLR**	0.8707	0.9471	0.8769	0.8367	0.9211
**GBDT-RF**	0.8817	0.9651	0.8691	0.9727	0.7855
**RLR-SVM-RF**	0.8817	0.9650	0.8691	0.9727	0.7855
**GBDT**	0.8770	0.9648	0.8636	0.9686	0.7792
**SVM-GBDT-RF**	0.8644	0.9656	0.8459	0.9793	0.7445
**SVM-GBDT**	0.8596	0.9662	0.8402	0.9750	0.7382
**SVM-RF**	0.8533	0.9645	0.8306	0.9828	0.7192
**RF**	0.8423	0.9636	0.8175	0.9697	0.7066
**SVM**	0.8391	0.9577	0.8104	0.9864	0.6877

Supplementary Table 7. Cohort Characteristics

	Braak Stage	N	Gender (M/F)	Age of death
**London (N=113)**	0-II	29	13/16	77.6(12.8)
	III-IV	18	7/11	88.5(5.2)
	V-VI	66	26/40	85.4(8.1)
**Mount Sinai (N=146)**	0-II	60	32/28	82(7.6)
	III-IV	42	12/30	88.8(6.6)
	V-VI	44	12/32	88.0(7.5)
**Arizona (N=302)**	0-II	61	40/21	80.3(8.2)
	III-IV	97	50/47	86.9(6.9)
	V-VI	144	63/81	82.3(8.5)
**ROS/MAP (N=739)**	0-II	151	75/76	83.6(7.2)
	III-IV	423	148/275	88.8(6.3)
	V-VI	165	46/119	89.8(5.2)

Supplementary Table 8. Number of training loci for each trait.

Outcome	Outcome Type	# CpGs sites
Amyloid	Pathologic, IHC	1,706
Braak Staging	Pathologic, Silver Stain	2,086
CERAD	Pathologic, Silver Stain	2,473
Cognitive Decline Trajectory	Clinical	2,374
Global Pathology	Pathologic, Silver Stain	3,039
Neurofibrillary Tangles	Pathologic, IHC	3,181

Supplementary Table 9. Demographic information of the sequenced samples and un-sequenced samples from original EWAS ROS/MAP cohort.

	Sequenced (N=150)	Un-sequenced (N=589)
**Male**	46	223
**Female**	104	366
**Age of death**	87.569 (6.408)	88.096 (6.738)
**Education**	16.080 (3.639)	16.487 (3.566)
**Amyloid**	3.372 (3.992)	3.498 (3.619)
**Braak Staging**	3.433 (1.297)	3.414 (1.258)
**CERAD**	2.407 (1.193)	2.299 (1.148)
**Cognitive Decline Trajectory**	-0.026 (0.107)	-0.030 (0.107)
**Global Pathology**	0.693 (0.660)	0.705 (0.615)
**Neurofibrillary Tangles**	7.481 (9.637)	6.102 (7.599)

Supplementary Table 10. Summary of feature categories used in the machine learning model. (* indicates new added features compared to features used in DIVAN [1].

Category	Feature
DNase	153
FAIRE	31
TF	571
Histone	1,002
RNA Pol II	49
*Total RNA-seq	243
*ATAC-seq	66
*WGBS	127
*Functional	14
**Total**	**2,256**

Reference:

1. Chen, L., Jin, P. & Qin, Z.S. DIVAN: accurate identification of non-coding disease-specific risk variants using multi-omics profiles. *Genome Biol* **17**, 252 (2016).
