# Supplementary material for: A machine learning approach to brain epigenetic analysis reveals kinases associated with Alzheimer’s disease
Source: Nat Commun. 2021 Jul 22;12:4472. doi: 10.1038/s41467-021-24710-8 (PMC8298578; doi:10.1038/s41467-021-24710-8)
Supplement: Supplementary file 3 — Description of Additional Supplementary Files [file 41467_2021_24710_MOESM3_ESM.pdf]

Description of Additional Supplementary Files

File Name: Supplementary Data 1
Description: Genes overlapped with the top 100 loci predicted by EWASplus to have the highest overall prediction scores for AD.

File Name: Supplementary Data 2
Description: Top 60 selected features for Beta-Amyloid (ROS/MAP).

File Name: Supplementary Data 3
Description: Top 60 selected features for Braak Staging (ROS/MAP).

File Name: Supplementary Data 4
Description: Top 60 selected features for CERAD (ROS/MAP).

File Name: Supplementary Data 5
Description: Top 60 selected features for Cognitive Decline Trajectory (ROS/MAP).

File Name: Supplementary Data 6
Description: Top 60 selected features for Global Pathology (ROS/MAP).

File Name: Supplementary Data 7
Description: Top 60 selected features for Neurofibrillary Tangles (ROS/MAP)

File Name: Supplementary Data 8
Description: Top 60 selected features for Braak Staging (London).

File Name: Supplementary Data 9
Description: Top 60 selected features for Braak Staging (Arizona).

File Name: Supplementary Data 10
Description: Top 60 selected features for Braak Staging (Mount Sinai).

File Name: Supplementary Data 11
Description: The list of primers for targeted bisulfite sequencing.
