# Supplementary material for: A machine learning approach to brain epigenetic analysis reveals kinases associated with Alzheimer’s disease
Source: Nat Commun. 2021 Jul 22;12:4472. doi: 10.1038/s41467-021-24710-8 (PMC8298578; doi:10.1038/s41467-021-24710-8)
Supplement: Supplementary file 5 — Reporting Summary [file 41467_2021_24710_MOESM5_ESM.pdf]

nature research

Corresponding author(s): Xiaobo Sun, Thomas S. Wingo, Zhaohui Qin

Last updated by author(s): Jun 10, 2021

Reporting Summary

Nature Research wishes to improve the reproducibility of the work that we publish. This form provides structure for consistency and transparency in reporting. For further information on Nature Research policies, see our Editorial Policies and the Editorial Policy Checklist.

Statistics

For all statistical analyses, confirm that the following items are present in the figure legend, table legend, main text, or Methods section.

n/a	Confirmed	
☐	☒	The exact sample size (n) for each experimental group/condition, given as a discrete number and unit of measurement
☒	☐	A statement on whether measurements were taken from distinct samples or whether the same sample was measured repeatedly
☐	☒	The statistical test(s) used AND whether they are one- or two-sided<br>*Only common tests should be described solely by name; describe more complex techniques in the Methods section.*
☒	☐	A description of all covariates tested
☐	☒	A description of any assumptions or corrections, such as tests of normality and adjustment for multiple comparisons
☐	☒	A full description of the statistical parameters including central tendency (e.g. means) or other basic estimates (e.g. regression coefficient) AND variation (e.g. standard deviation) or associated estimates of uncertainty (e.g. confidence intervals)
☐	☒	For null hypothesis testing, the test statistic (e.g. F, t, r) with confidence intervals, effect sizes, degrees of freedom and P value noted<br>*Give P values as exact values whenever suitable.*
☒	☐	For Bayesian analysis, information on the choice of priors and Markov chain Monte Carlo settings
☒	☐	For hierarchical and complex designs, identification of the appropriate level for tests and full reporting of outcomes
☒	☐	Estimates of effect sizes (e.g. Cohen's d, Pearson's r), indicating how they were calculated

Our web collection on statistics for biologists contains articles on many of the points above.

Software and code

Policy information about availability of computer code

Data collection	No custom software was used in the raw data collection step.
Data analysis	The full data analysis pipeline can be found from this open source github repository: https://github.com/xsun28/EWASplus/tree/remastered.<br>A detailed step-by-step README is provided in the github page as well.<br>Software versions: Python 3.6, R 3.4, pandas 0.25.0, scikit-learn 0.19.1, numpy 1.13.3, pyliftover 0.4, hyperopt 0.2.2, GenomicRanges 1.34.0, GenomicRanges 1.34.1, RColorBrewer 1.1, ggplot2 3.3.1, CMplot 3.6.2.<br>All software and packages listed above are open source and freely available.

For manuscripts utilizing custom algorithms or software that are central to the research but not yet described in published literature, software must be made available to editors and reviewers. We strongly encourage code deposition in a community repository (e.g. GitHub). See the Nature Research guidelines for submitting code & software for further information.

Data

Policy information about availability of data

All manuscripts must include a data availability statement. This statement should provide the following information, where applicable:
- Accession codes, unique identifiers, or web links for publicly available datasets
- A list of figures that have associated raw data
- A description of any restrictions on data availability

1) Raw data: alignment data from ENCODE (https://www.encodeproject.org/), publicly available annotation scores such as: CADD (https://cadd.gs.washington.edu/), GWAVA (https://www.sanger.ac.uk/sanger/StatGen_Gwava) , EIGEN (http://www.columbia.edu/~ii2135/eigen.html), GenoCanyon (http://genocanyon.med.yale.edu/) and etc.
2) Prediction scores of six AD-related traits for all CpG loci (N=26573858) across the whole human genome (size: ~3GB)

3) Processed features data for all CpG loci (N=26,573,858) across the whole human genome (size: ~400GB)
4) the result of protein-protein interaction and pathway analyses
5) EWAS summary statistics for ROS/MAP
6) Targeted Bisulfite Sequencing data

Notes:
1) is publicly available data and the urls are provided as above.
2) , 3) and 4) are now available at https://figshare.com/collections/Dataset_collection_of_EWASplus/5430207
5) and 6) are available through Synapse (https://www.doi.org/10.7303/syn25781400). Researchers who are interested in downloading the data must have a free Synapse account.

Field-specific reporting

Please select the one below that is the best fit for your research. If you are not sure, read the appropriate sections before making your selection.

- [x] Life sciences
- [ ] Behavioural & social sciences
- [ ] Ecological, evolutionary & environmental sciences

For a reference copy of the document with all sections, see nature.com/documents/nr-reporting-summary-flat.pdf

Life sciences study design

All studies must disclose on these points even when the disclosure is negative.

Sample size	EWAS differential methylation analysis with 450K array methylation data: 717 ROS/MAP participants<br>Targeted Bisulfite Seqeucncing: 150 ROS/MAP participants
Data exclusions	1) EWAS differential methylation analysis excludes non-Caucasian subjects to avoid spurious findings due to differences in population structure, we focused the EWAS analysis focused on individuals of European descent who make up the largest proportion of the available methylation data. This led to 22 individuals who were not of European descent being excluded from the EWAS analysis.<br>2) For the methylation data, we removed probes annotated to multiple chromosomes or the X or Y chromosomes by Illumina, probes that cross-hybridize with other probes due to sequence similarity, probes with a detection p-value > 0.01 in any sample, probes without a CpG, and probes that overlap with a common SNP.<br>All exclusion criteria were pre-established.
Replication	Randomly picked samples were subject to targeted bisulfite sequencing only once.
Randomization	The demographic statistics comparison for the randomly selected samples (N=150) and non-selected samples(N=589) about the key phenotypes (gender, beta-amyloid, braak staging, cognitive decline trajectory and etc.) are shown in the Supplementary Table 9. No significant difference in those key phenotypes is observed.
Blinding	The investigators were blind to the sample information when performing targeted bisulfite sequencing. The blinding is not applicable for other analysis included in this paper (e.g., EWAS).

Reporting for specific materials, systems and methods

We require information from authors about some types of materials, experimental systems and methods used in many studies. Here, indicate whether each material, system or method listed is relevant to your study. If you are not sure if a list item applies to your research, read the appropriate section before selecting a response.

Materials & experimental systems

n/a	Involved in the study
☒	☐ Antibodies
☒	☐ Eukaryotic cell lines
☒	☐ Palaeontology and archaeology
☒	☐ Animals and other organisms
☐	☒ Human research participants
☒	☐ Clinical data
☒	☐ Dual use research of concern

Methods

n/a	Involved in the study
☒	☐ ChIP-seq
☒	☐ Flow cytometry
☒	☐ MRI-based neuroimaging

Human research participants

Policy information about studies involving human research participants

Population characteristics	After excluding non-Caucasian subjects, ROS/MAP participants (N=717) have a high degree of education (median of 16 years) and are predominantly women (63%). Their ages at death range from 66 to 108 years, with a median age at death of 88 years. Additionally, 42% of these individuals had a clinical diagnosis of AD at death.
Recruitment	The ROS project, established in 1994, recruited Catholic priests, nuns, and brothers from 40 groups in 12 states who were at least 55 years of age and free of known dementia at the time of enrollment. The MAP cohort, established in 1997, recruited people primarily from retirement facilities in the Chicago area who were at least 53 years of age and free of known dementia at the time of enrollment. All participants in ROS and MAP sign an informed consent agreeing to annual detailed clinical evaluations and cognitive tests, and the rate of follow-up exceeds 90%. Similarly, participants in both cohorts signed an Anatomical Gift Act donating their brains at the time of death. The overall autopsy rate exceeds 85%. We consider it unlikely that there is any recruitment bias that would materially affect out results.
Ethics oversight	ROS and MAP related research projects were approved by the Institutional Review Boards of Rush University Medical Center. The full information on the approval of the study protocol is provided in the "Cohorts" section of the "Method" part.

Note that full information on the approval of the study protocol must also be provided in the manuscript.
